# Tilorone attenuates high-fat diet-induced hepatic steatosis by enhancing BMP9-Smad1/5/8 signaling

**DOI:** 10.1007/s11357-025-01685-8

**Published:** 2025-05-27

**Authors:** Barnabas Horvath, Judit Halasz, Norman Noel Tanner, Zoltan Marton Kohler, Gyorgy Trencsenyi, Laszlo Juhasz, Laszlo Rovo, Andras Kiss, Aniko Keller-Pinter

**Affiliations:** 1 Department of Biochemistry, Albert Szent-Gyorgyi Medical School, Centre of Excellence for Interdisciplinary Research, Development and Innovation, University of Szeged, Szeged, Hungary; 2 Department of Pathology, Forensic and Insurance Medicine, Semmelweis University, Budapest, Hungary; 3 Department of Medical Imaging, Divison of Nuclear Medicine and Translation Imaging, Faculty of Medicine, University of Debrecen, Debrecen, Hungary; 4 Institute of Surgical Research, Albert Szent-Gyorgyi Medical School, University of Szeged, Szeged, Hungary; 5 Department of Oto- Rhino- Laryngology and Head and Neck Surgery, University of Szeged, Szeged, Hungary; 6 Department of Internal Medicine, Albert Szent-Gyorgyi Medical School, University of Szeged, Szeged, Hungary

**Keywords:** Liver, High-fat diet, Metabolic dysfunction-associated steatotic liver disease, Tilorone, Bone morphogenetic protein, Insulin resistance, Mitochondria, PPARy

## Abstract

The prevalence of metabolic dysfunction-associated steatotic liver disease (MASLD) is rapidly increasing and is caused by excessive fat deposition in the liver due to non-alcoholic factors. Aging is a major risk factor for the development and progression of MASLD. In this study, we investigated the metabolic effects of tilorone, a synthetic small molecule, in a high-fat diet (HFD) mouse model, with a focus on the liver function and signaling. We demonstrate that tilorone attenuated HFD-induced steatosis by restoring bone morphogenetic protein 9 (BMP9)-Smad1/5/8 signaling and upregulating peroxisome proliferator-activated receptor gamma (PPARγ) expression. Tilorone reduced HFD-induced increases in body weight, adipose tissue and liver weight, and blood glucose levels, and improved glucose tolerance in HFD mice. PET/MRI imaging demonstrated enhanced ^18^FDG (^18^F-fluoro-2-deoxyglucose) uptake in liver, skeletal muscle, adipose tissue, and myocardium of tilorone-treated HFD animals. Histological analysis showed that tilorone reduced the HFD-induced diffuse, macrovesicular steatosis (S3/3), and machine learning-based image analysis revealed a decrease in lipid droplet size and lipid content. HFD caused the disappearance of liver glycogen, but tilorone increased glycogen levels. High-resolution respirometry indicated that tilorone reduced HFD-induced increases in mitochondrial complex II-linked oxidative phosphorylation and complex IV activity. These findings revealed the beneficial effects of tilorone on HFD and highlight its therapeutic potential in MASLD, particularly given that tilorone is a synthetic small molecule and can be administered orally. Further studies are required to explore its clinical application.

**Supplementary Information:**

The online version contains supplementary material available at 10.1007/s11357-025-01685-8.

## Introduction

Age-related diseases have a global impact as they are the leading cause of death [1]. The aging liver is affected by several conditions that can cause liver function to decline. Metabolic dysfunction-associated steatotic liver disease (MASLD), formerly known as non-alcoholic fatty liver disease (NAFLD), is the most common liver disease in Western industrialized countries with a growing incidence worldwide, making it a global health problem. Aging is associated with the severity and poor prognosis of various liver diseases, including MASLD [2]. Aging increases the risk of developing MASLD, and accelerated biological aging promotes the development of MASLD [3]. The incidence of MASLD increases with age. In 2015, the median age of the MASLD population was 50 years, and research predicts that this will increase to 55 years by 2030 [4].

MASLD is frequently associated with type 2 diabetes mellitus (T2DM) and obesity, with an overall prevalence of 25% in the general population and more than 60% in patients with diabetes [5]. MASLD is defined based on the evidence of liver steatosis accompanied by at least one of the following conditions: obesity, diabetes, or metabolic dysregulation [6]. In the early stage of MASLD, in a simple fatty liver, accumulation of lipids without inflammation and liver cell damage can be observed. The more severe form of MASLD is metabolic dysfunction-associated steatohepatitis (MASH), in which fat accumulation causes inflammation and liver cell damage, which can progress further to cirrhosis. The accumulation of aging cells drives liver steatosis, and liver cell aging is also closely related to the progression of MASLD [4]. People over 50 years of age of are more susceptible to the progression of MASLD than younger people [7].

MASLD is defined as hepatic triglyceride accumulation of more than 5% that is not caused by excessive alcohol consumption [8]. Under healthy conditions, only small amounts of fatty acids are stored as lipids in cytosolic lipid droplets in the liver [9]. When the balance between the formation and mobilization of lipid droplets and the secretion of lipoproteins and/or bile acids is disturbed, then MASLD develops due to the large amount of lipid droplet accumulation.

Bone morphogenetic proteins (BMPs) are members of the transforming growth factor β superfamily. More than 30 BMPs have been identified, 15 of which are found in mammals [10]. BMPs exert their effects through the Smad signaling pathway. Upon binding to their heterodimeric receptors, phosho-Smad1/5/8 forms a complex with Smad4 and translocates to the nucleus [11]. Several studies described the role of BMP family members in whole-body metabolism and their crucial roles in liver development and homeostasis. For example, BMP9 plays a key role in lipid and glucose metabolism and was shown to be effective in reducing body weight, liver mass, and the development of steatosis in mice on a high-fat diet (HFD) [12]. Moreover, BMP9 knockout mice exhibit liver steatosis via the downregulation of peroxisome proliferator-activated receptor alpha (PPARalpha) expression [13]. BMP4 also reduces body weight and serum triglyceride levels and reduces the risk of developing MASH [14]. BMP2 expression was increased in the liver of MASH patients [15], and BMP6 deficient mice develop increased hepatic inflammation and fibrosis [16].

Tilorone dihydrochloride, a small molecular weight synthetic compound, was discovered in the 1970 s [17]. The discovery of new drugs to treat viral infections had been in great demand since the 1970 s, and the testing of the antiviral activity of tilorone was considered [18] and has been successfully tested against Ebola, influenza [19], or SARS-CoV-2 [20]. In addition, antipyretic and anti-inflammatory [21], antitumor [22], neuroprotective [23], and antifibrotic properties in the case of pulmonar fibrosis [24] and cardiac fibrosis [25] have also been proven. Notably, tilorone increases BMP2,4, and 7 levels in airway epithelial cells [24] and BMP2,4,7, and 14 levels in C2 C12 myoblasts [26].

The increasing global prevalence of MASLD makes the discovery and testing of new therapeutic agents particularly important. In general, the prevalence of MASLD is 15–30% in adults; however, the prevalence of MASLD [1] and the risk of liver fibrosis increases with age [27], with nearly half of people aged 80 years and older having MASLD [28]. Given the role of BMPs in MASLD and the BMP-inducing effect of tilorone, we aimed to investigate the effect of tilorone on liver steatosis in vivo in a HFD mouse model.

## Materials and methods

### Experimental animals

Twelve- to fourteen-week-old male C57BL/6 mice (Akronom Kft, Budapest, Hungary) were used for the experiments. The animals were housed under conventional conditions at 22 ± 2 °C with 55 ± 10% humidity and artificial lighting with a circadian cycle of 12 h. Food and drinking water were freely accessible to all animals. Laboratory animals were kept and treated in accordance with all applicable sections of the Hungarian laws and animal welfare directions and regulations of the European Union. The investigation was approved by the Regional Animal Research Ethics Committee of Csongrad County, Hungary (number: XV./2662/2021).

### Experimental design

**Fig. 1:**
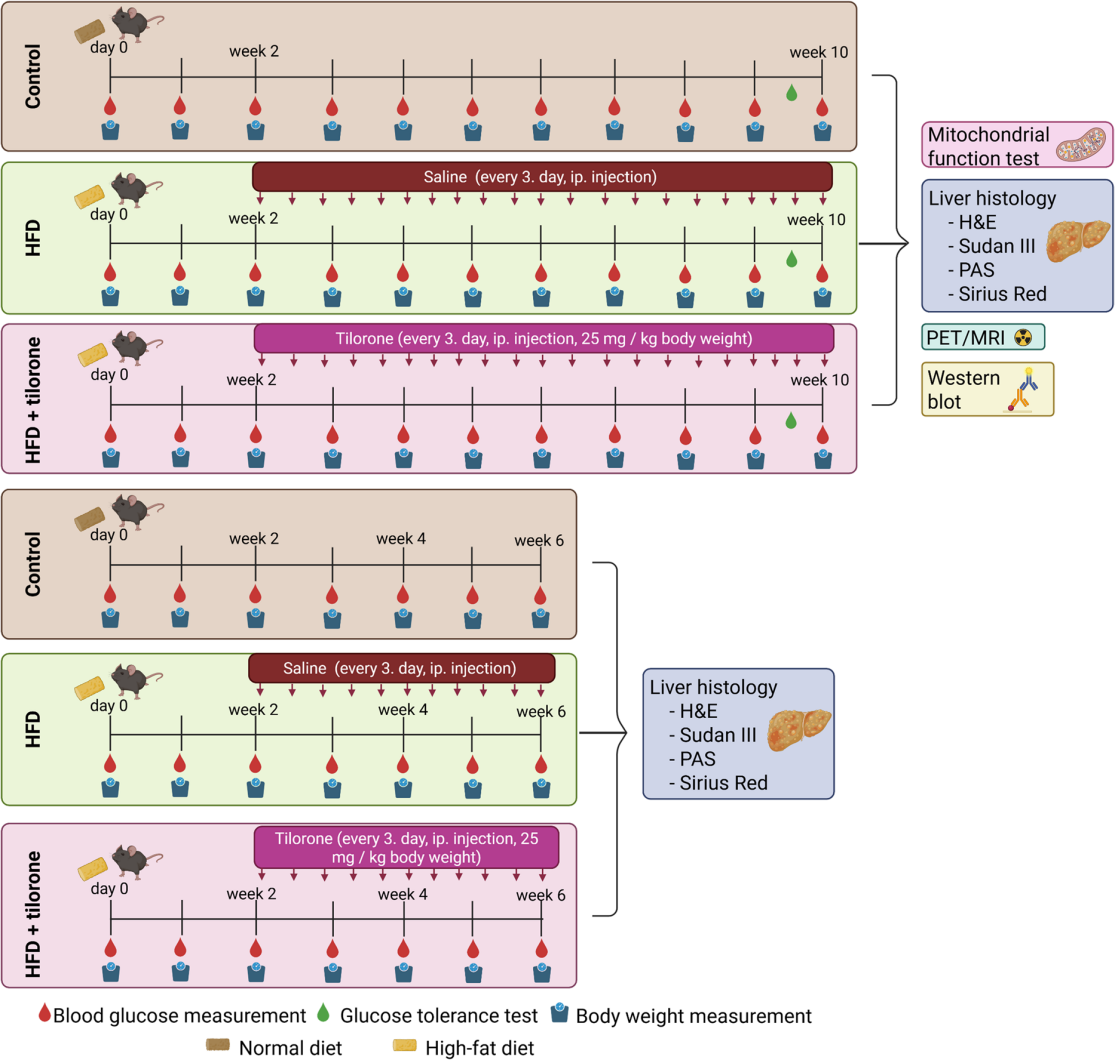
Shematic summary of experimental design. Ip., intraperitoneal; HFD, high-fat diet

For the 10-week experimental period, mice (*n* = 30) were randomly divided into three groups: (1) in the control group, mice were kept on normal food (LT/R rodent feed, Sinbad Kft. Gödöllő, Hungary); (2) in the HFD (high-fat diet) group, mice were kept on HFD including 20% protein; 36% fat and 36.7% carbohydrate (HF260 rodent feed, SAFE®, Augy, France; detailed composition is shown in Supplementary Fig. 1) for 10 weeks and were treated with intraperitoneal saline (25 µl saline/kg body weight, every third day during weeks 3–10); and (3) in the HFD + tilorone group, animals were treated with intraperitoneal tilorone (25 mg tilorone/kg body weight dissolved in saline, every third day during weeks 3–10) (Fig. 1). The blood glucose and body mass values were measured every week. On the 9 th week, an intraperitoneal glucose tolerance test was performed. One set of animals (*n* = 4–5/each group) also underwent in vivo PET/MRI at the end of the experimental period. To euthanize the animals, sodium pentobarbital was overdosed (Euthasol, 200 mg/kg, ip.; Produlab Pharma b.v., Raamsdonksveer, The Netherlands). The hindlimb muscles, liver, heart, abdominal fat, and kidneys were then removed and weighed, and the tibia was also isolated. Liver samples were immediately subjected to high-resolution respirometry analysis, and tissue samples were also embedded for histological analysis and frozen for further investigations. For histological analysis of the liver, one set of animals (*n* = 3–4) in each group was terminated after 6 weeks. (Fig. 1).

### Blood glucose measurement and intraperitoneal glucose tolerance test

Before the blood glucose measurements, mice were fasted for 16 h and had free access to water. Mice were anaesthetized with 60 mg/kg body weight of thiopental sodium, and blood glucose measurements were performed from the distal tail vein.

The intraperitoneal glucose tolerance test was performed at week 9. Before measurement, the mice fasted for 16 h and had free access to water. The measurement of baseline blood glucose was followed by intraperitoneal injection of D-glucose (2 mg glucose/1 g body weight), and blood glucose was determined from distal tail vein at 0, 15, 30, 45, 60, 90, and 120 min. All blood glucose measurements were performed with the Accu-Check blood glucose monitoring system (Roche; Basel, Switzerland).

### Measurement of ^18^F-fluoro-2-deoxyglucose (^18^FDG) uptakein vivo

To monitor the uptake of the radioactive glucose analog ^18^FDG, PET/MRI measurements were performed on HFD and tilorone treated mice. After the measurements, HFD mice were injected intraperitoneally with tilorone (25 mg/kg body weight), and the treatment was repeated 3 days after the first injection, and new PET/MR scans were performed on the day after the second injection of tilorone (HFD + 2 × tilorone group).

For PET/MRI measurements, mice were anaesthetized with 3% Forane using a dedicated small animal anesthesia device and were injected with 10.2 ± 0.9 MBq of ^18^FDG in 100 µl saline through the lateral tail vein. Fifty minutes after radioactive injection, whole-body PET/MR scans were performed under isoflurane anesthesia using the preclinical nanoScan 1 T PET/MR device (Mediso LTD., Hungary). After the image reconstruction and PET image analysis, the standardized uptake value (SUV) was calculated using the following formula: SUV = [ROI activity (MBq/mL)]/[injected activity (MBq)/animal weight (g)].

### Assessment of mitochondrial oxygen consumption using high-resolution respirometry

Mitochondrial oxygen consumption (O_2_ flux) was evaluated in liver homogenate (10% w/v; 50 µL/chamber) using High-Resolution FluoRespirometry (Oxygraph-2 k, Oroboros Instruments, Innsbruck, Austria). Approximately 200–300 mg of liver samples were cut with a sharp scissor and homogenized with a Potter–Elvehjem tissue grinder in Mir05 medium. After stabilization of baseline respiration, complex I-linked oxidative phosphorylation (OXPHOS I) was measured in the presence of complex I-linked substrates (10 mM glutamate and 2 mM malate) and ADP (2.5 mM). Rotenone (Rot; 0.5 µM) was used to (a) inhibit complex I and (b) assess complex II-linked oxidative phosphorylation (OXPHOS II) in the presence of succinate (S; 10 mM) and adenylate. After inhibition of complex III (antimycin A; 2.5 µM), complex IV (CIV) respiratory activity was measured with ascorbate (2 mM) and artificial substrate TMPD (0.5 mM). Ascorbate was added before TMPD to avoid uncontrollable autoxidation of the electron donor. Sodium azide (NaN_3_; 100 mM) was finally administered to block complex IV-linked mitochondrial respiration. Measurements were carried out in a Mir05 respiration medium under continuous magnetic stirring (750 rpm) at 37 °C. The DatLab software (Oroboros Instruments, Innsbruck, Austria) was used for online display, respirometry data acquisition, and analysis.

### Liver histology

The lipid content of the liver samples was visualized on 4-µm thick frozen sections with Sudan III staining. Sirius Red staining was used to investigate the signs of fibrosis in liver samples following HFD. PAS (periodic acid-Schiff) reaction was applied to determine the presence of tissue glycogen. To prove that the PAS positivity represents glycogen and not other polysaccharides or other substances, we also performed PAS staining after digestion with diastase. Liver sections stained with hematoxylin and eosin, Sudan III, Sirius Red, and PAS, respectively, were scanned with a digital camera (PANNORAMIC™ Digital Slide Scanners; 3D Histech).

To evaluate of the levels of steatosis in samples stained with hematoxylin and eosin, we applied the steatosis staging scale (S0–S3) used in human pathological diagnostics (S0: less than 5% of the hepatocytes contain lipid droplets; S1: 5–33% of the hepatocytes contain lipid droplets; S2: 33–66% of the hepatocytes contain lipid droplets; and S3: more than 66% of the hepatocytes contain lipid droplets). The quality of the results was verified by two independent pathologists.

### Determination of liver glycogen

In addition to PAS staining, liver glycogen was measured as glucose residues after acidic hydrolysis by a standard enzymatic assay. Briefly, following cryogenic milling, the samples (10–15 mg) were digested in 2 M HCl (250 µL HCl/10 mg muscle tissue) at 100 °C during continuous shaking (1000 rpm; Thermoshaker, ThermoFisher Scientific, Waltham, Massachusetts USA) for 1.5 h. After lysis, the samples were cooled to room temperature and neutralized by adding of equal amount of 2 M NaOH. Thereafter, the samples were centrifuged for 10 min at 14,000 × g. The concentration of glucose was determined from the supernatant by glucose hexokinase assay (Siemens Healthcare Diagnostics inc., ADVIA® Chemistry, Tarrrytown, New York, USA) according to the manufacturer’s instructions.

### Image analysis of lipid droplets

For the analysis of the lipid content of liver samples, the numbers and areas of individual lipid droplets were measured on Sudan III stained slides with NIS-Elements BR software (Nikon Instruments Inc., Melville, NY, USA). This software utilizes machine learning to accurately select droplets with lipid content and determine the area and number of selected droplets. We examined the entire section and normalized the data to the number of nuclei.

### Western blotting

Liver samples were homogenized in a buffer (50 mM Tris–HCL pH = 7.6; 100 mM NaCl; 10 mM EDTA) supplemented with 1 mM sodium fluoride and 1 mM sodium orthovanadate and a protease inhibitor cocktail (#P8340; Sigma-Aldrich, St. Louis, MO, USA). After homogenization, the samples were centrifuged at 16,000 × g for 10 min at 4 °C to remove debris, and then protein levels were determined using the bicinchoninic acid method (#23,227; ThermoFisher Scientific, Waltham, MA, USA). Samples containing equal amounts of proteins were separated via sodium dodecyl sulfate–polyacrylamide gel electrophoresis and transferred to Protran nitrocellulose membrane (GE Healthcare, Buckinghamshire, UK). Then, the membranes were blocked in Tris buffered saline containing 5% skimmed milk and 0.1% Tween-20 (Sigma-Aldrich) for 1 h at room temperature and were incubated at 4 °C overnight with the following rabbit polyclonal primary antibodies: phospho-Smad1/5/8 (Smad1 [Ser463/465]/Smad5 [Ser463/465]/Smad9 [Ser465/467]; #AB-3848-J; Sigma-Aldrich); Smad1/5/8 (#56,656; Novus Biologicals; Centennial, CO, USA); PPARγ (#2435; Cell Signaling Technology; Danvers; MA; USA); PGC-1α (#2178; Cell Signaling Technology); TOM20 (#42,406; Cell Signaling Technology); BMP6 (#55,421–1-AP; Proteintech; Rosemont; IL, USA); BMP9 (#17,769–1-AP; Proteintech); and GAPDH (#2188; Cell Signaling Technology). Subsequently, membranes were incubated with the horseradish peroxidase-conjugated anti-rabbit IgG secondary antibody (#P0448) from DAKO. The peroxidase activity was developed using the enhanced chemiluminescence (#K-12045, Advansta, San Jose, CA, USA) procedure, and the chemiluminescence signal was recorded using X-ray films (Agfa, Mortsel, Belgium). On the scanned X-ray films, bands were selected with uniformed square ROIs (region of interest) using the BioRad Quantity One Analysis Software (Bio-Rad, Hercules, CA, USA). Then, using the “Volume Analysis Report” function, we obtained the intensity of the selected area normalized to the background.

### Statistical analysis

Statistical significance between groups was analyzed using one-way analysis (ANOVA) of variance followed by Sidak’s post-hoc test. GraphPad Prism 9 (GraphPad Software Inc., La Jolla, CA, USA) was used for graphing and statistical analyses. The data are expressed as the mean ± SEM or the median (horizontal line in the box) with the 25 th (lower whisker) and 75 th (upper whisker) percentiles plotted on box plots; *p* < 0.05 denoted statistical significance.

## Results

### Tilorone treatment decreased body mass and blood glucose level and improved glucose tolerance in HFD mice

**Fig. 2:**
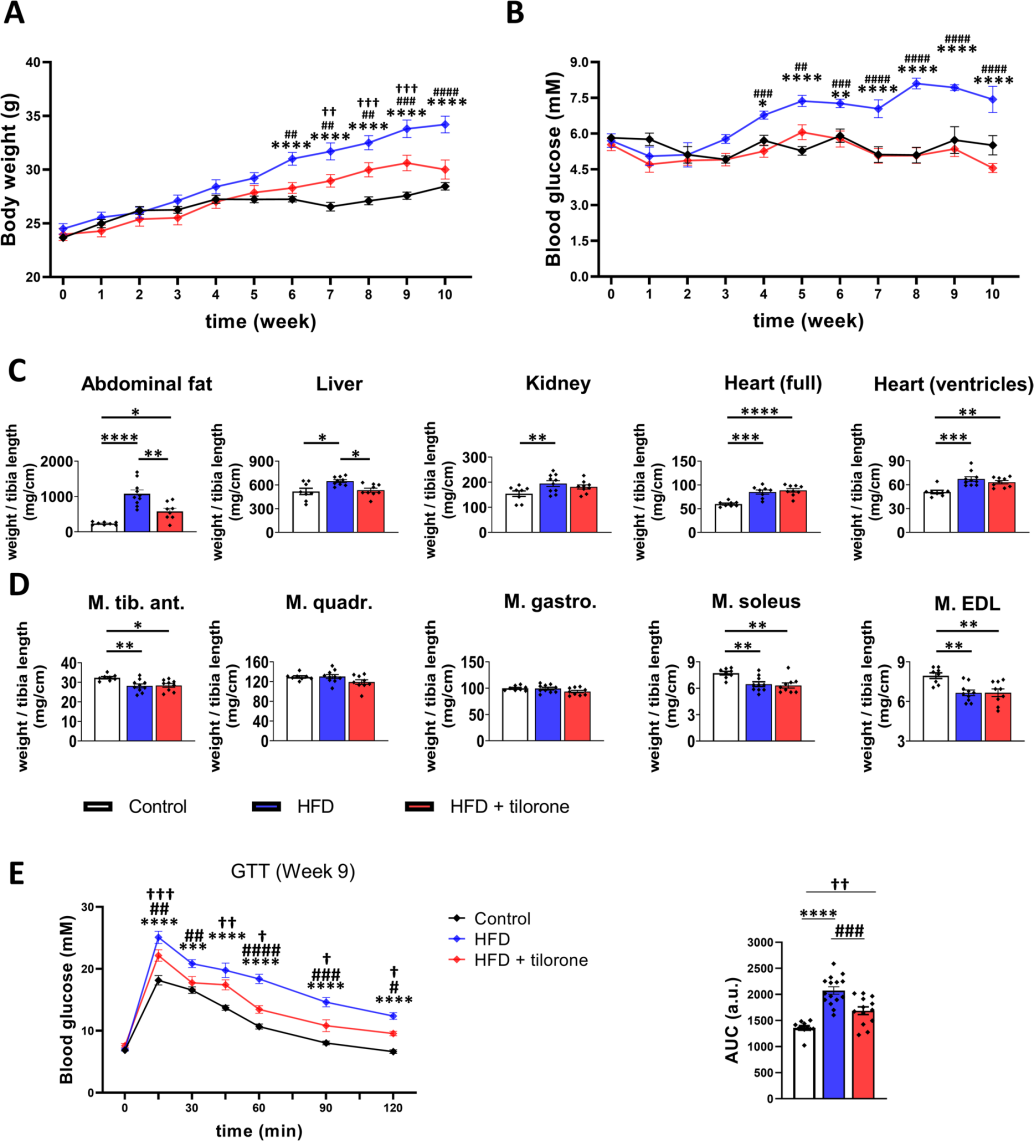
Effect of high-fat diet (HFD) and tilorone treatment on glucose metabolism. **A** Body weights, **B** blood glucose levels, **C** organ and **D** muscle weights of control, HFD, and tilorone-treated HFD mice are shown. **E** Glucose tolerance test (GTT) and area under the curve (AUC) values are shown. Data are reported as mean ± SEM; *n* = 8–15; control vs. HFD: *p < 0.05; **p < 0.01; ***p < 0.001; ****p < 0.0001; HFD vs. HFD + tilorone: #*p* < 0.05; ##*p* < 0.01; ###*p* < 0.001; ####*p* < 0.0001; control vs. HFD + tilorone: †*p* < 0.05; ††*p* < 0.01; †††*p* < 0.001; a.u.: arbitrary unit

The HFD mouse model is widely used to study T2DM and MASLD. During the experimental period, the body weight of HFD mice increased continuously (all animals had the same body mass before the experiments). Notably, tilorone treatment reduced the HFD-induced increase in body mass. At the end of the 10-week experimental period, the weights of the tilorone treated HFD mice were comparable to those of the controls (Fig. 2A). Fasting blood glucose levels were measured weekly during the 10-week experimental period. HFD increased blood glucose levels and persistently higher levels were detected than those of other groups (Fig. 2B). Importantly, tilorone normalized the bood glucose levels of HFD animals.

Notably, tilorone administration reduced the HFD-induced increases in the weights of liver and abdominal fat. Kidney and heart weights also increased in HFD mice, but tilorone did not cause any changes (Fig. 2C). We also investigated the effects of HFD and tilorone on hindlimb muscle weights. The weights of M. tibialis anterior, M. soleus and M. extensor digitorum longus decreased after HFD, and tilorone treatment could not compensate for these changes. The weights of M. quadriceps and M. gastrocnemius did not show any differences (Fig. 2D).

To determince the effect of tilorone on tissue glucose uptake of HFD mice, we performed an intraperitoneal glucose tolerance test at the end of the 9 th week. HFD resulted in decreased glucose tolerance and increased area under the curve value (Fig. 2E). Importantly, tilorone treatment improved the glucose tolerance of HFD mice (Fig. 2E).

### Tilorone administration increased ^18^FDG uptakein vivo

**Fig. 3:**
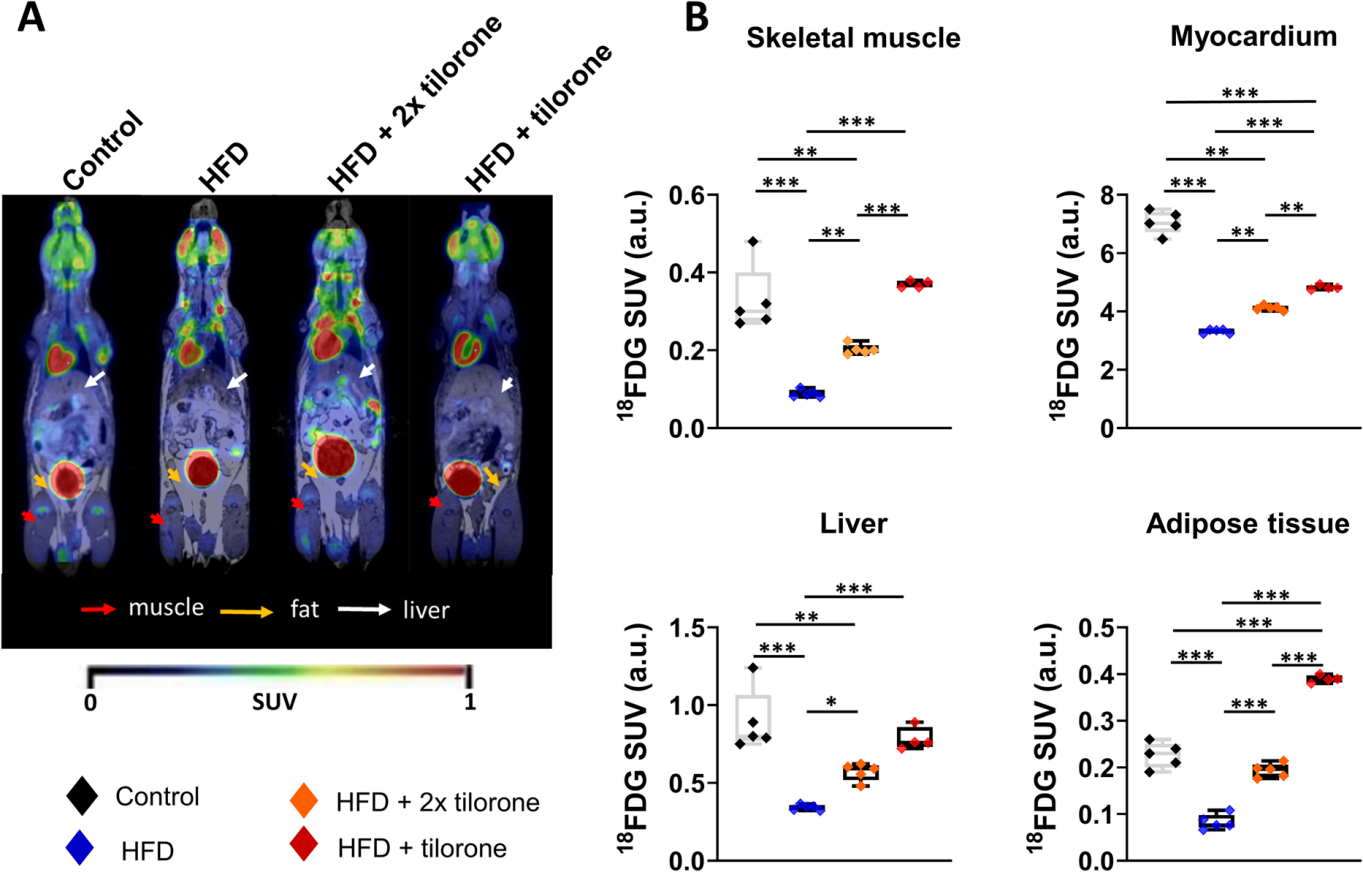
In vivo ^18^F-fluoro-2-deoxyglucose (^18^FDG) uptake in C57BL/6 mice. **A** Representative decay-corrected ^18^FDG-PET/MR images of control, HFD (10-week), HFD animals treated with two injections of tilorone at the end of experimental period (HFD + 2 × tilorone), and HFD animals treated with tilorone for 8 weeks (HFD + tilorone). **B** Quantitative analysis of ^18^FDG uptake of selected tissues (skeletal muscle, myocardium, liver, fat). The box plots demonstrate the median (horizontal line in the box) and the 25 th (lower whisker) and 75 th (upper whisker) percentiles. *n* = 4–5; *p < 0.05; **p < 0.01; ***p < 0.001; a.u.: arbitrary unit

The glucose tolerance test demonstrated that HFD reduced the glucose tolerance, and tilorone increased the whole body glucose uptake in HFD mice. Next, we evaluated the distribution of radiolabelled glucose representing the glucose uptake in different tissues using small-animal PET/MRI. By the qantitative analysis of decay-corrected ^18^FDG-PET images, we found significant differences in the SUV mean of the selected organs after tracer injection (Fig. 3A). Tilorone treatment (HFD + tilorone group) resulted in ~ 3.5–fourfold increase in radiolabelled glucose uptake of skeletal muscle, liver, adipose tissue, and ~ 1,fivefold increase in myocardium (Fig. 3B).

Notably, the short-term tilorone treatment also had significant effects on ^18^FDG uptake. The HFD animals treated with two injections of tilorone at the end of the experimental period (HFD + 2 × tilorone) also showed a significant increase in ^18^FDG uptake in all investigated organs (skeletal muscle, liver, adipose tissue, and heart) (Fig. 3D).

### Tilorone reduced the HFD-induced increases in complex II-linked oxidative phosphorylation and compex IV activity

**Fig. 4:**
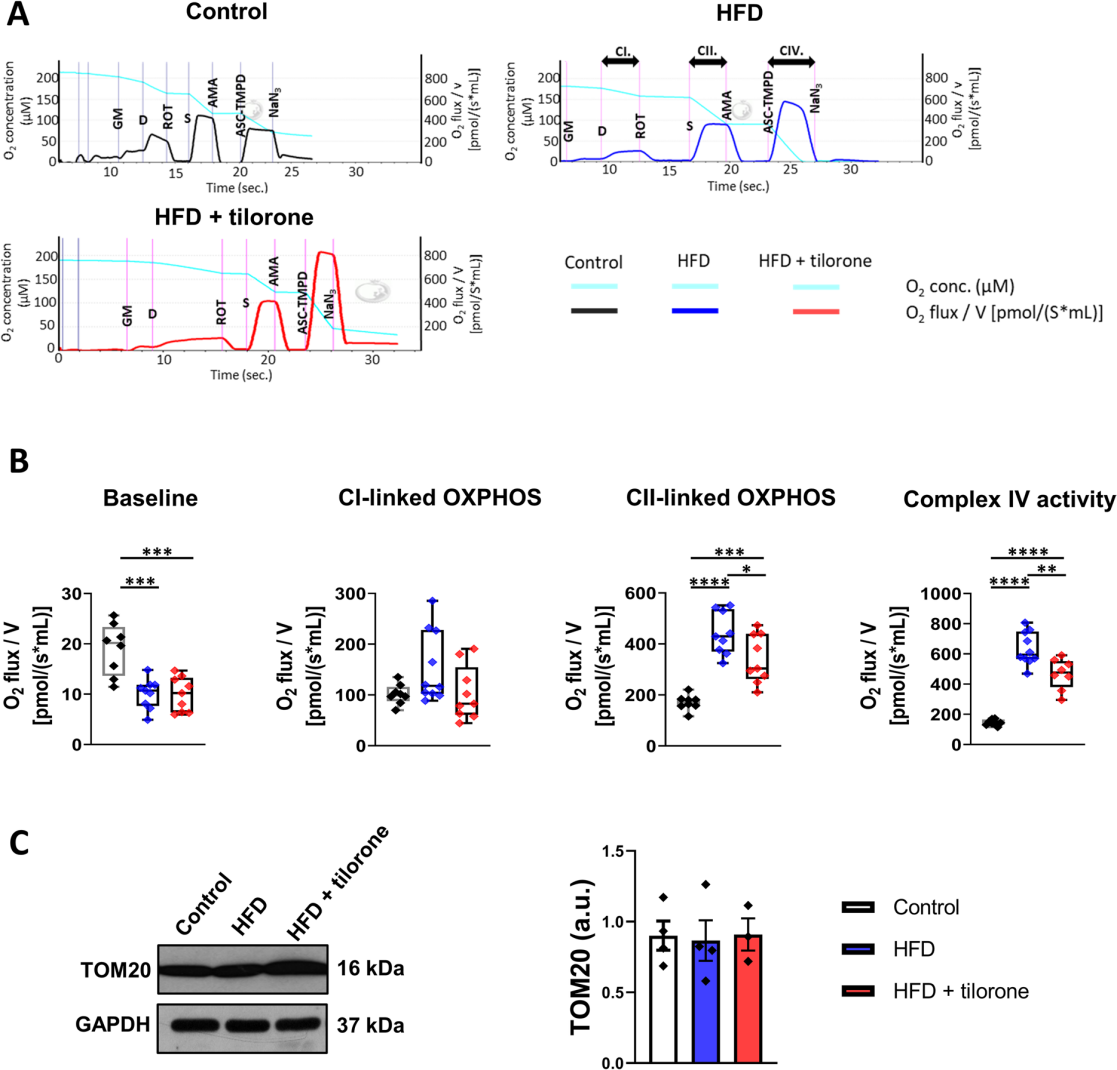
High-resolution respirometry analysis of liver samples. **A** Control, HFD, and tilorone-treated HFD C57BL/6 mouse liver samples were analyzed with high-resolution respirometry. Representative graphs of the respirometry analysis. **B** Baseline, complex I-linked oxidative phosphorylation (OXPHOS), complex II-linked OXPHOS values, and complex IV activity were measured. The box plots demonstrate the median (horizontal line in the box) and the 25 th (lower whisker) and 75 th (upper whisker) percentiles; *n* = 6–7; *p < 0.05; **p < 0.01; ***p < 0.001; ****p < 0.0001. GM, glutamate and malate; D, ADP; ROT, rotenone; S, succinate; AMA, antimycin A; ASC-TMPD, ascorbate/TMPD; NaN_3_, sodium azide; CI., complex I-linked OXHOS; CII., complex II-linked OXPHOS; CIV., complex IV activity. **C** Representative western blot depicts the expression of TOM20. Quantification of the results is shown. GAPDH was used as a loading control; protein levels were normalized to GAPDH, *n* = 3–4 in each group; a.u.: arbitrary unit

Mitochondria play a key role in oxidative stress and calcium and cellular respiration homeostasis [29]. PET/MRI results showed that tilorone treatment increased the tissue glucose uptake in HFD mice. To investigate what the increased amount of glucose was used for, we performed high-resolution respirometry assays on fresh liver tissue samples (Fig. 4A).

Baseline (routine) respiration decreased in HFD mice and tilorone treated HFD group compared to control group. Neither HFD alone nor tilorone treatment of HFD animals had any effect on complex I-linked oxidative phosphorylation (Fig. 4B). After the addition of succinate, information on complex II-linked oxidative phosphorylation was obtained. Based on our results, HFD increased the complex II-linked oxidative phosphorylation, and tilorone administration significantly decreased it in HFD animals. Next, ascorbate was used to determine the activity of complex IV. Tilorone treatment reduced the HFD-induced increase in the complex IV activity in HFD animals, similarly in case of complex II-linked oxidative phosphorylation (Fig. 4B).

Next, we compared the mitochondrial number of the groups based on the expression levels of TOM20, a mitochondrial membrane protein. We demonstrated that neither HFD alone nor tilorone treatment of HFD animals resulted in changes in mitochondrial number (Fig. 4C).

### Tilorone normalized pSmad1/5/8 levels and the expression of BMP9 and PPARγ in HFD animals

The key molecule in the BMP signaling pathway is the Smad1/5/8 transciption factor. Phosphorylated Smad1/5/8 forms dimers and translocates to the nucleus to regulate gene expression. Our results show that HFD induced a decrease in pSmad1/5/8 level which increased to the control level after tilorone treatment. The increased levels of pSmad1/5/8 indicate echanced BMP signaling after tilorone treatment. Since we found increased pSmad/5/8 levels, indicating enhanced BMP signaling, we next tested the expression levels of BMP6 and BMP9 proteins, which play important roles in liver homeostasis. Consistent with pSmad1/5/8 levels, HFD decreased the expression of BMP6 and BMP9 compared to the control group. Notably, tilorone treatment normalized the HFD-induced decrease in BMP9 level. However, BMP6 expression did not change after tilorone treatment of HFD animals.

**Fig. 5:**
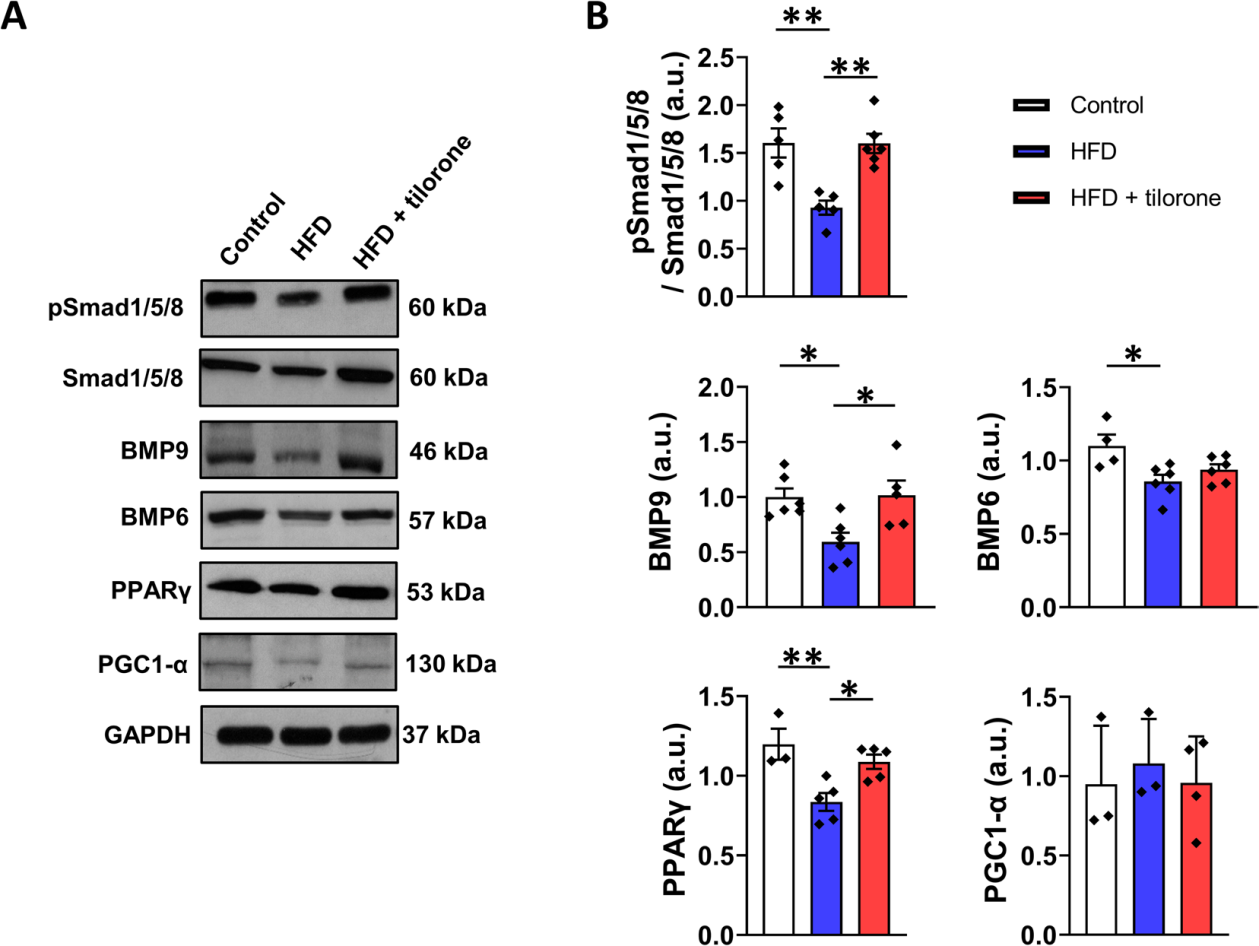
Effect of high-fat diet (HFD) and tilorone administration on the levels of signaling molecules. **A** Representative western blots depict the levels of phospho-Smad1/5/8 (Ser463/465/467), Smad1/5/8, BMP9, BMP6, PPARγ, and PGC1-α in control, HFD (10-week) liver samples, and following tilorone administration. GAPDH was used as a loading control. **B** Quantification of the western blot results of **A**, protein levels were normalized to GAPDH. Data are reported as the mean + SEM (*n* = 3–5); *p < 0.05; **p < 0.01; a.u.: arbitrary unit

Peroxisome proliferator-activated receptor gamma (PPARγ) functions as a transcription factor and plays a role in lipid and glucose metabolism. Importantly, the PPARγ-PGC1α(PPARγ coactivator 1 alpha) axis regulates mitochondrial biogenesis. Based on our western blot results, the expression level of PPARγ was decreased by HFD and tilorone administration increased it. However, neither HFD alone nor tilorone treatment resulted in changes in PGC-1α protein levels (Fig. 5).

### Tilorone treatment reduced lipid content of HFD liver

**Fig. 6:**
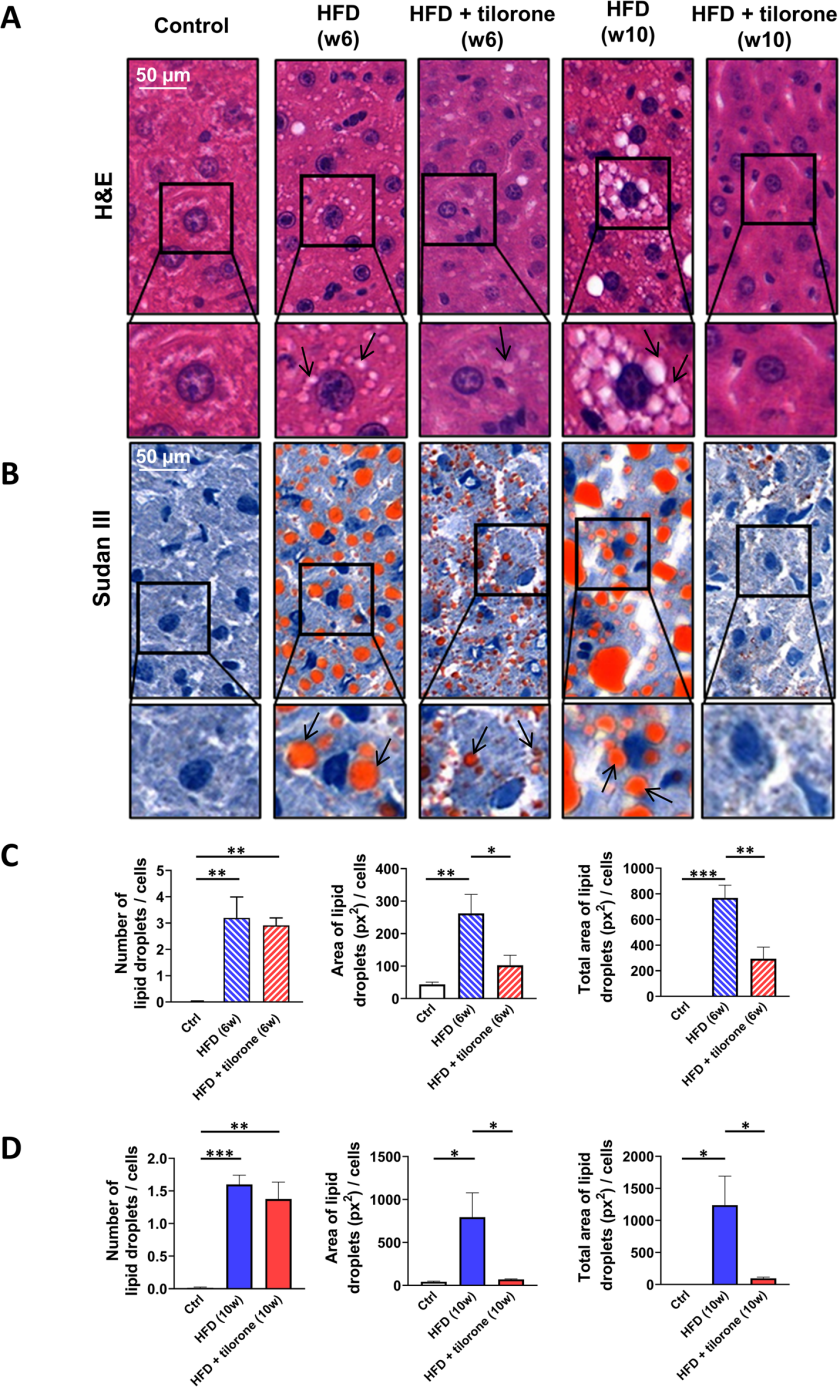
Tilorone administration decreases the lipid droplet size and lipid content in the high-fat diet (HFD) liver. Representative images of **A** hematoxylin and eosin and **B** Sudan III stained control, HFD (6-week and 10-week experimental period), and tilorone-treated HFD (6-week and 10-week experimental period) liver samples. Number of lipid droplets, area of the individual droplets, and total area of the lipid droplets of the hepatocytes were analyzed following **C** 6-week and **D** 10-week experimental period. Arrows show lipid droplets. *N* = 3–4 samples in each group were analyzed. Data are reported as mean + SEM; *n* = 3–4; *p < 0.05; **p < 0.01; ***p < 0.001

MASLD and MASH are important complication of obesity, T2DM, and hyperlipidaemia. Hematoxylin and eosin-stained liver specimens were analyzed to detect the rate of steatosis in different groups of mice. In the control group, the hepatocytes were characterized by moderately broad eosinophilic cytoplasm, and the nuclei showed mild size variability. We did not find any lipid droplets in the control liver. However, we detected diffuse, dominantly small droplets of macrovesicular steatosis (S3/3) in the HFD liver samples (Fig. 6A).

Sudan III-stained liver samples (Fig. 6B) were analyzed to determine the number and area of lipid droplets in different groups of mice after the 10-week experimental period. To determine the dynamics in liver droplet formation, we also analyzed liver samples from one set of animals in each group at week 6. Machine learning-based image analysis of the samples showed that the total areas of lipid droplets, representing the lipid content of the liver, increased after HFD in both the 6-week and 10-week experimental groups (Fig. 6C, D). Notably, more lipid droplets were formed after 6 than 10 weeks of HFD (Fig. 6C, D); however, after 10 weeks, larger droplets were detected, resulting in higher lipid content of the liver.

Importantly, the average area of the individual lipid droplets decreased after tilorone treatment during both the 6- (Fig. 6C) and 10-week experimantal periods (Fig. 6D); however, the number of these droplets did not decrease significantly. The total area of the lipid droplets per cell also decreased, representing the decreased lipid content of the tilorone-treated HFD liver (Fig. 6C, D). The prolonged use of tilorone until the end of week 10 decreased the size of lipid droplets to the level of the control group (Fig. 6D). Overall, tilorone treatment successfully reduced the size of lipid droplets and the lipid droplet content of liver cells.

Histopathological analysis of hematoxylin and eosin-stained liver specimens revealed no evidence of inflammation in control animals with preserved hepatic architecture (Fig. 6A, Supplementary Fig. 2). In contrast, all HFD animals exhibited focal inflammatory infiltrates (Supplementary Fig. 2) regardless of the feeding duration (6 or 10 weeks). The inflammatory foci were predominantly composed of lymphocytes, with a smaller proportion of neutrophils, and were scattered throughout the lobules. In several foci, the inflammatory lesions resulted in small spotty necrosis. No inflammatory infiltration was observed within the portal areas. These findings suggest an early onset of hepatic inflammation under HFD conditions. Similarly, animals receiving HFD combined with tilorone treatment showed consistent focal inflammatory foci, with a distribution and cellular composition similar to the HFD group. The degree of inflammation was comparable to that in the HFD group, indicating that tilorone did not significantly alter the inflammatory status of the liver over the time period studied. No ballooned hepatocytes containing Mallory-Denk bodies were observed, and no classic steatohepatitis was identified. In certain inflammatory foci, apoptotic bodies were discernible, suggesting ongoing cell turnover and tissue damage. Overall, both HFD and HFD + tilorone groups demonstrated a mild to moderate degree of hepatic inflammation, primarily focal in distribution, whereas control animals remained largely free of inflammatory changes (Supplementary Fig. 2).

### Effect of tilorone on glycogen content of the liver

**Fig. 7:**
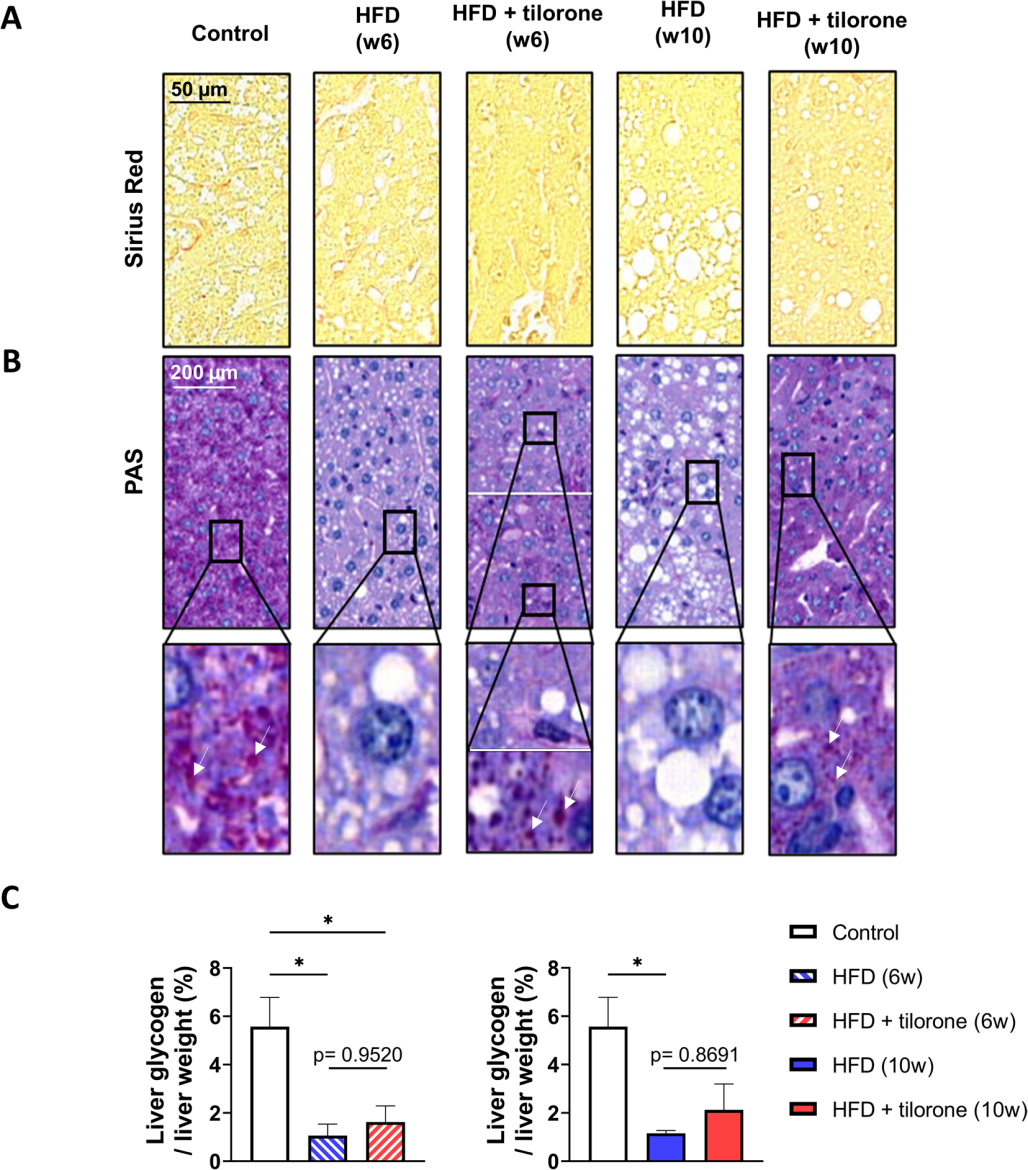
Tilorone affects liver glycogen. Representative images of **A** Sirius Red and **B** Periodic Acid Schiff (PAS)-stained liver samples of control, HFD (6-week and 10-week experimental period), and tilorone-treated HFD mice (6-week and 10-week experimental period). Arrows show glycogen granules. **C** Glycogen content of control, HFD (6-week and 10-week experimental period), and tilorone-treated HFD (6-week and 10-week experimental period) liver samples was quantified. Data are reported as the mean ± SEM (*n* = 3–4); *p < 0.05

To assess fibrosis induced by HFD, we performed Sirius Red staining on liver specimens. The results show that no fibrosis was developed in the liver samples of mice during the experimental period (Fig. 7A).

Since tilorone increased ^18^FDG uptake in the liver, next, we determined the glycogen content of the liver samples. PAS staining was used to evaluate tissue glycogen levels. Histological results showed that tissue glycogen was almost disappeared in HFD mice compared to the control group. Tilorone treatment increased the glycogen content of liver samples in HFD animals, and glycogen droplets could be observed (Fig. 7B). The distribution of these glycogen droplets in the liver was not uniform after 6 weeks. However, a more visible increase in glycogen was only observed after week 10, with a homogenous distribution. We also performed PAS staining in combination with diastase (Supplementary Fig. 3) to prove that the observed polysaccharide is glycogen. Based on these results, the increased glucose uptake in the liver of tilorone treated HFD mice was used, at least in part, for glycogen formation.

To quantify the amount of the liver glycogen, we measured the glucose residues after hydrolysis of glycogen. HFD decreased the glycogen content of the liver compared to control group in both 6- and 10-week experimental period. Tilorone administration did not significantly increase glycogen content over the period studied; only an increasing trend was observed (Fig. 7C).

## Discussion

Aging affects liver function and increases the risk of developing MASLD, which is the most common liver disease worldwide. Because the prevalence of MASLD increases with age, and MASLD-related complications may represent an important source of morbidity and healthcare utilization, this disease should be addressed in the elderly population [30]. In recent decades, considerable efforts have been made to better understand the pathogenesis and to identify therapeutic targets. Due to the worldwide prevalence of MASH, numerous clinical trials have been initiated to develop potential therapeutic agents; however, because of the heterogeneity of the disease, only few drugs are available [31]. This makes the discovery and testing of new agents particularly important.

The development of MASLD is common among people with diabetes; 70% of people with T2DM develop fatty liver [32]. Several studies have described that BMPs play a key role in the regulation of glucose and lipid metabolism, and BMP4 has been shown to significantly inhibit the development of steatosis [33]. Here, we found that HFD decreased Smad1/5/8 phosphorylation and tilorone administration increased it to the levels of control.

Tilorone is known primarily for its antiviral effects and commercially available in some countries [19]. Leppäranta and collagues demonstrated increased expression of BMP2 and BMP7 in epithelial cells [24]. Earlier we showed that tilorone increased BMP2,4,7,14 levels in C2 C12 myoblasts and increased the glucose uptake of myoblasts and myotubes [26]. In line with these results, tilorone increased the activation of Smad1/5/8, indicating increased BMP signaling and increased glucose uptake of different tissues (liver, adipose tissue, skeletal muscle, and heart) in a HFD mice model. In addition, tilorone improved blood glucose levels and glucose tolerance, suggesting favorable effects on glucose metabolism.

We have successfully modeled pathological obesity and demonstrated the weight loss effects of tilorone in an HFD model. Increased body weight and blood glucose levels were detected in HFD animals during 10-week experiments. We also observed a significant increase in adipose tissue weight after HFD, which was reduced by tilorone. In clinical practice, semaglutide, a GLP-1 (glucagon-like peptide-1) agonist, is used as an anti-obesity drug for weight loss because of its ability to stimulate insulin secretion in a glucose-dependent manner [34]. Because of its different mechanism of action to tilorone, it would be interesting to test its effect in combination with tilorone in the future.

Several studies have already shown that HFD worsens glucose tolerance [35]. Our results also confirmed that HFD caused a decrease in glucose tolerance, and tilorone treatment improved it. A previous study has shown that obesity and sedentary lifestyle can cause muscle atrophy [36]. Consistent with these results, after 10 weeks, we detected a significant reduction in weights of tibialis anterior, soleus and extensor digitorum longus muscles; however, tilorone treatment could not compensate for this decrease.

**Fig. 8:**
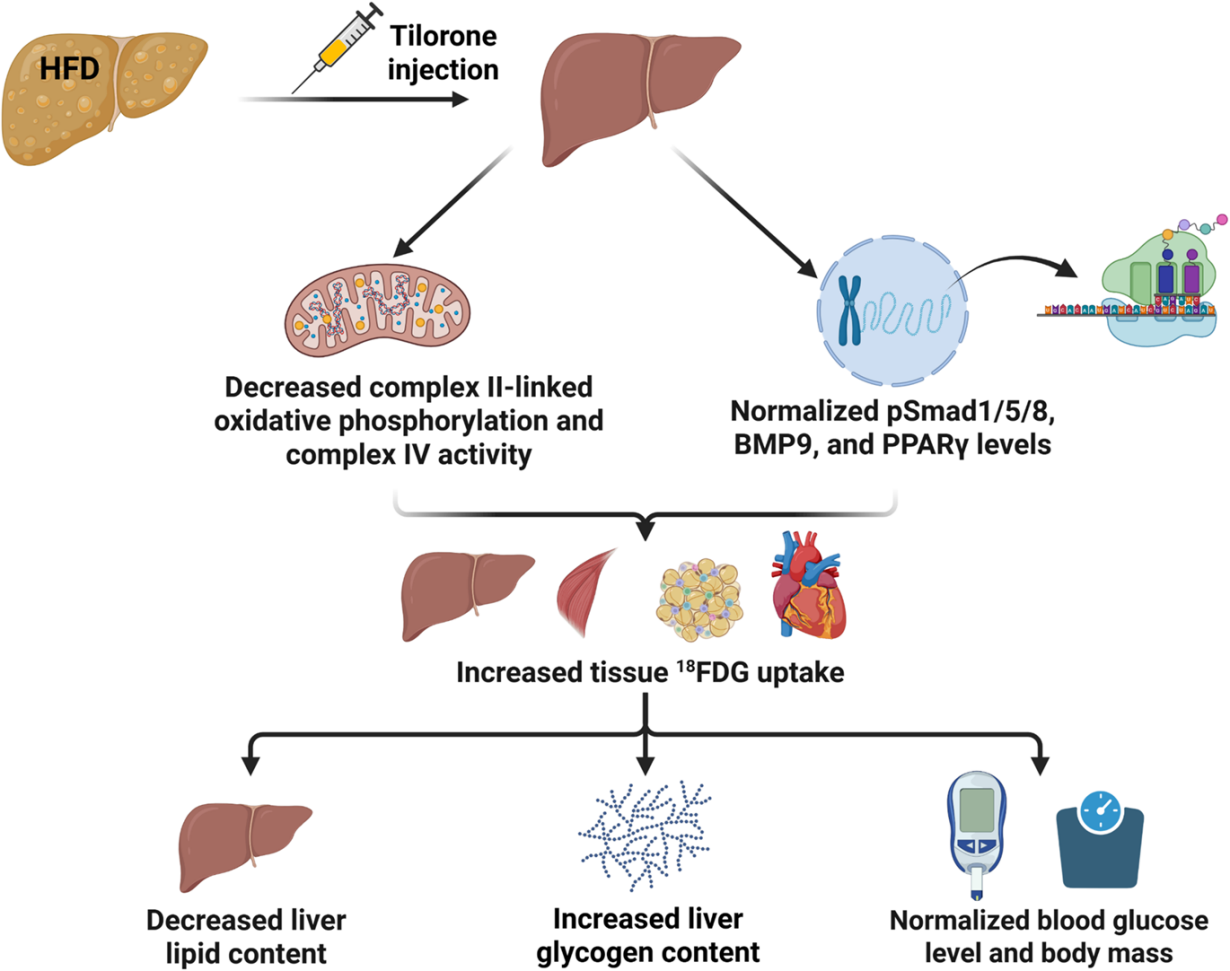
Summary of the results. After the 10 weeks tilorone treatment, the HFD-induced increases in complex II-linked oxidative phosphorylation and complex IV activity reduced and the HFD-induced decreases in BMP9, pSmad1/5/8, and PPARγ levels were normalized. PET/MRI showed increased tissue uptake of ^18^FDG. As a consequence, lipid content of the liver decreased, the glycogen content increased, and blood glucose level, body mass, and liver and adipose tissue weights were normalized. HFD, high-fat diet; BMP, bone morphogenetic protein; PPARγ, peroxisome proliferator-activated receptor gamma; ^18^FDG, ^18^F-fluoro-2-deoxyglucose

HFD induced steatosis and administration of tilorone affected the mitochondrial function. Tilorone reduced the HFD-induced increases in complex II-linked oxidative phosphorylation and compex IV activity in the liver. These changes can lead to decreased ATP production in HFD animals after tilorone treatment, and the consequently increased AMP/ATP ratio can lead to enhanced AMPK activity, resulting in increased liver glucose uptake, as observed on PET/MRI. Based on PAS staining, this increased ^18^FDG uptake by the liver was used for glycogen production (Fig. 8). PAS staining of liver samples clearly showed that liver glycogen disappeared in HFD animals and glycogen droplets were observed after tilorone treatment, which were more pronounced in the 10-week experimental group. However, this increase in glycogen, as measured by glucose residues, was not significant.

The earlier published mitochondrial effects of tilorone are in line with our recent observations. In C2 C12 myoblasts tilorone treatment decreased ATP-linked respiration and consequently increased the ^18^FDG uptake of the cells [26]. In addition, tilorone treatment did not lead to changes in the number of mitochondria in the HFD liver, but only affected mitochondrial function. This result is consistent with our previous study in myoblasts, where we described that tilorone did not affect the amount of mitochondria in C2 C12 cells [26].

The PET/MRI results demonstrated an increase in ^18^FDG uptake in the liver. Short-term tilorone administration, two tilorone injections, significantly increased liver glucose uptake, which was further increased in the group of animals receiving regular tilorone injections for 8 weeks. Importantly, both short-term and long-term administration of tilorone increased glucose uptake in skeletal muscle, adipose tissue, and the heart.

PPARγ regulates fatty acid gene expression and adipocyte differentation [37]. PPARγ plays a key role in lipid homeostasis and improves glucose uptake in T2DM [38]. As a result of the binding of BMPs to their receptors, phosphorylated Smad1/5/8 forms a complex with Smad4, translocates to the nucleus, and enhances PPARγ expression [11]. Our results show that PPARγ expression levels are decreased by HFD, but tilorone normalized PPARγ levels, glucose uptake, and the lipid content of the liver.

Both BMPs are important players in MASLD. HFD causes a decrease in BMP9 levels which contributes to the development of MASLD [39]. BMP9-knockout mice exhibit steatosis due to downregulated PPARα expression and reduced fatty acid oxidation [13]. Furthermore, in vitro, recombinant BMP9 treatment attenuates triglyceride accumulation [13]. Consistent with these results, we found that tilorone compensates for the HFD-induced decrease in BMP9 expression. Earlier sudy showed that BMP6 deficient mice develop hepatic inflammation [16]. Interestingly, we found that BMP6 expression was also decreased in HFD mice; however, tilorone did not increase BMP6 levels during the experimental period and the observed mild to moderate degree of hepatic inflammation in HFD animals did not disappear after tilorone treatment.

Clinical studies have found a correlation between reduced BMP9 levels and the development of MASLD [39]. Aging causes BMP9 levels to decrease, increasing the risk of developing MASLD [40]. In our experiment, tilorone treatment normalized the HFD-induced decrease in BMP9 levels. Since BMP9 levels decrease during aging [40], we hypothesize that tilorone treatment may also be effective in aged mice.

The transcription factor PGC-1α plays a central role in mitochondrial biogenesis [31]. Neither HFD nor tilorone treatment induced changes in PGC-1α expression levels. It is likely that a longer experimental period would result in significant differences between the groups. However, PGC-1α levels did not change in mice on a high-energy diet for 11 months [41]. A longer feeding period is also likely to be required for the development of liver fibrosis [42], as Sirius Red staining showed no evidence of fibrosis developing in the liver of HFD animals after 10 weeks.

HFD increases the number of lipid droplets in the livers of animals [43]. During HFD, lipid droplets were developed in the liver, leading to steatosis. We detected a significant increase in the number of lipid droplets in the liver after 6 weeks of HFD. Interestingly, fewer, but larger lipid droplets, were formed during the 10-week feeding period compared to 6-week, leading to an increase in liver lipid content. Deep learning-based image analysis of Sudan III stained samples clearly demonstrated that tilorone treatment decreased the lipid content of the liver of HFD animals. Tilorone reduced the area of these droplets, but we did not find significant changes in the numbers of droplets after tilorone administration. In line with the histological signs of steatosis observed, liver weight also increased in HFD animals and was reduced to the level of controls in the tilorone-treated group.

In conclusion, in this study we demonstrated that tilorone treatment improved steatosis and glucose tolerance and decreased body mass and adipose tissue weight accumulation in HFD mice (Fig. 8). MASDL is the most common liver disease worldwide, and efforts are needed to address the challenges of an aging population and to find treatments for the different stages of MASDL. Given that tilorone is a synthetic, small molecule with multiple beneficial effects and can be administered orally, the use of tilorone for the treatment of steatosis might be clinically considered. As the antifibrotic effect of tilorone has already been demonstrated in pulmonary and cardiac fibrosis, it may be worth considering the administration of tilorone in liver fibrosis which might progress to cirrhosis. Further studies are warranted to explore its potential clinical application.

## Supplementary Information

Below is the link to the electronic supplementary material.

Supplementary file1 (PNG 1087 KB)

Supplementary file2 (PNG 2092 KB)

Supplementary file3 (PNG 2009 KB)

## Data Availability

The datasets generated and analyzed during the current study are available from the corresponding author on reasonable request.

## Abbreviations

MASLD: Metabolic dysfunction-associated steatotic liver disease
NAFLD: Non-alcoholic fatty-liver disease
MASH: Metabolic dysfunction-associated steatohepatitis
HFD: High-fat diet
BMP: Bone morphogenetic protein
^18^FDG: ^18^F-fluoro-2-deoxyglucose
T2DM: Type 2 diabetes mellitus
