# Supplementary figures and images for: Tilorone attenuates high-fat diet-induced hepatic steatosis by enhancing BMP9-Smad1/5/8 signaling
Source: GeroScience. 2025 May 27;48(1):1097–114. doi: 10.1007/s11357-025-01685-8 (PMC12972450; doi:10.1007/s11357-025-01685-8)

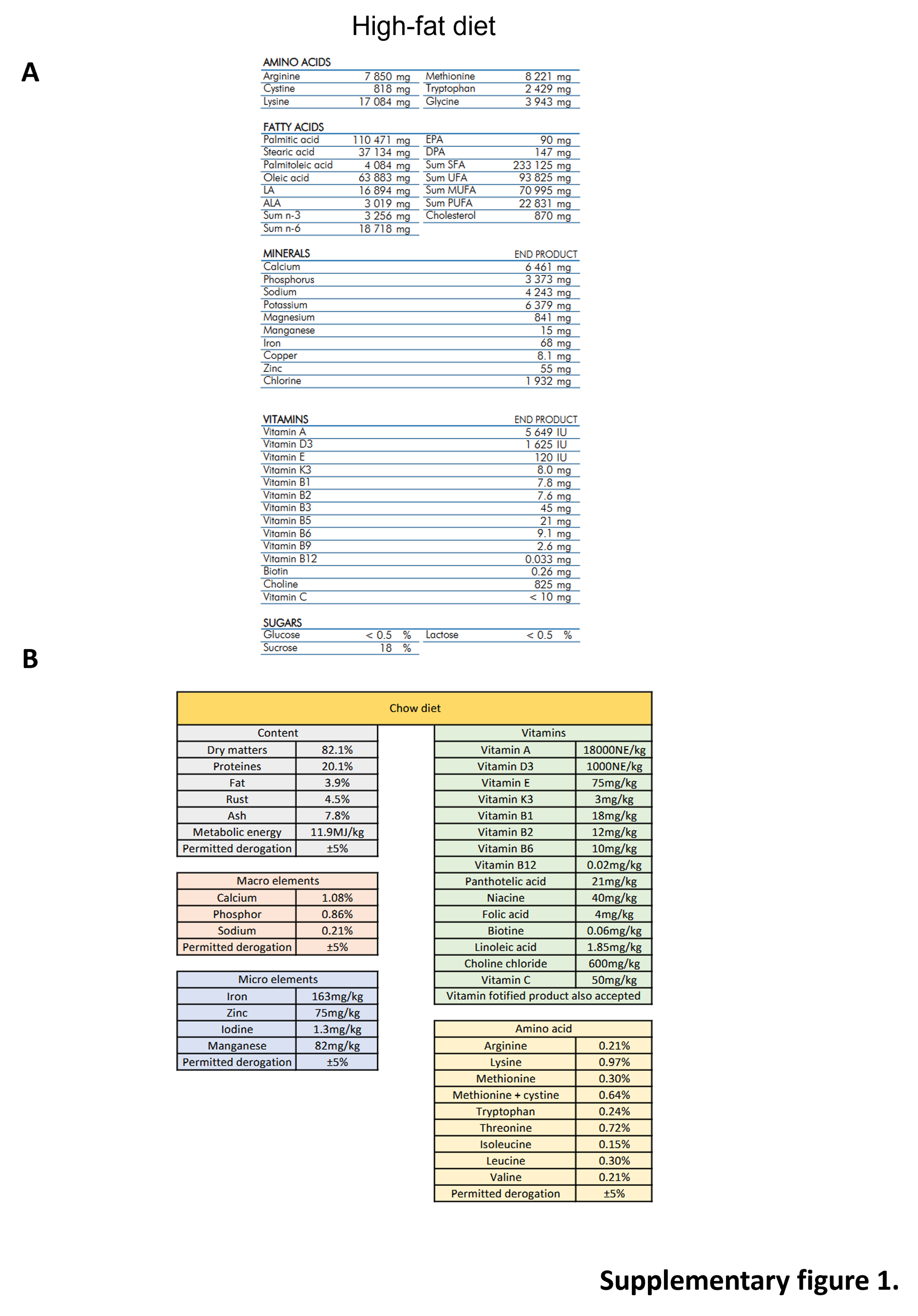

Supplement: Supplementary file 1 — Supplementary file1 (PNG 1087 KB) [file 11357_2025_1685_MOESM1_ESM.png]

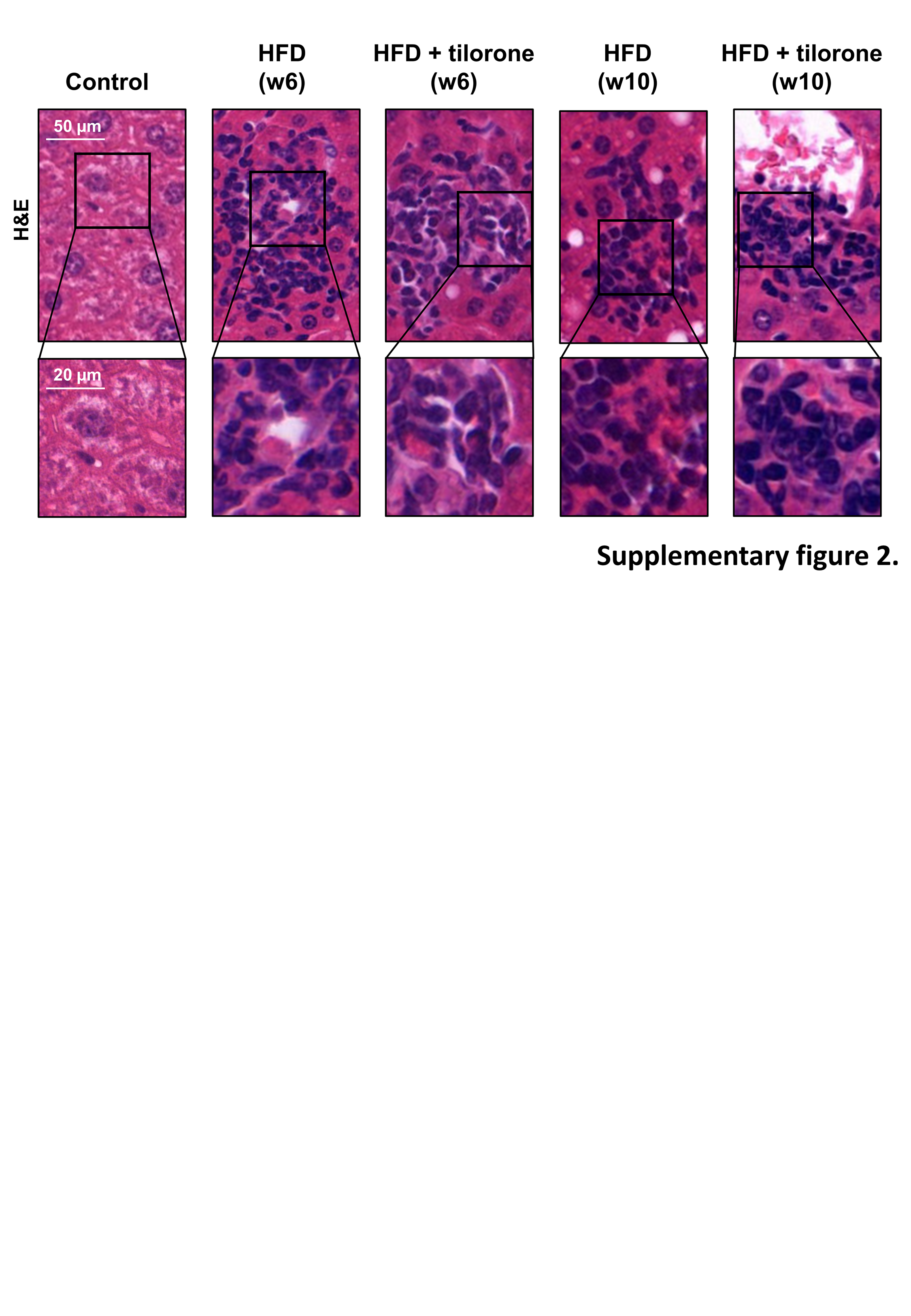

Supplement: Supplementary file 2 — Supplementary file2 (PNG 2092 KB) [file 11357_2025_1685_MOESM2_ESM.png]

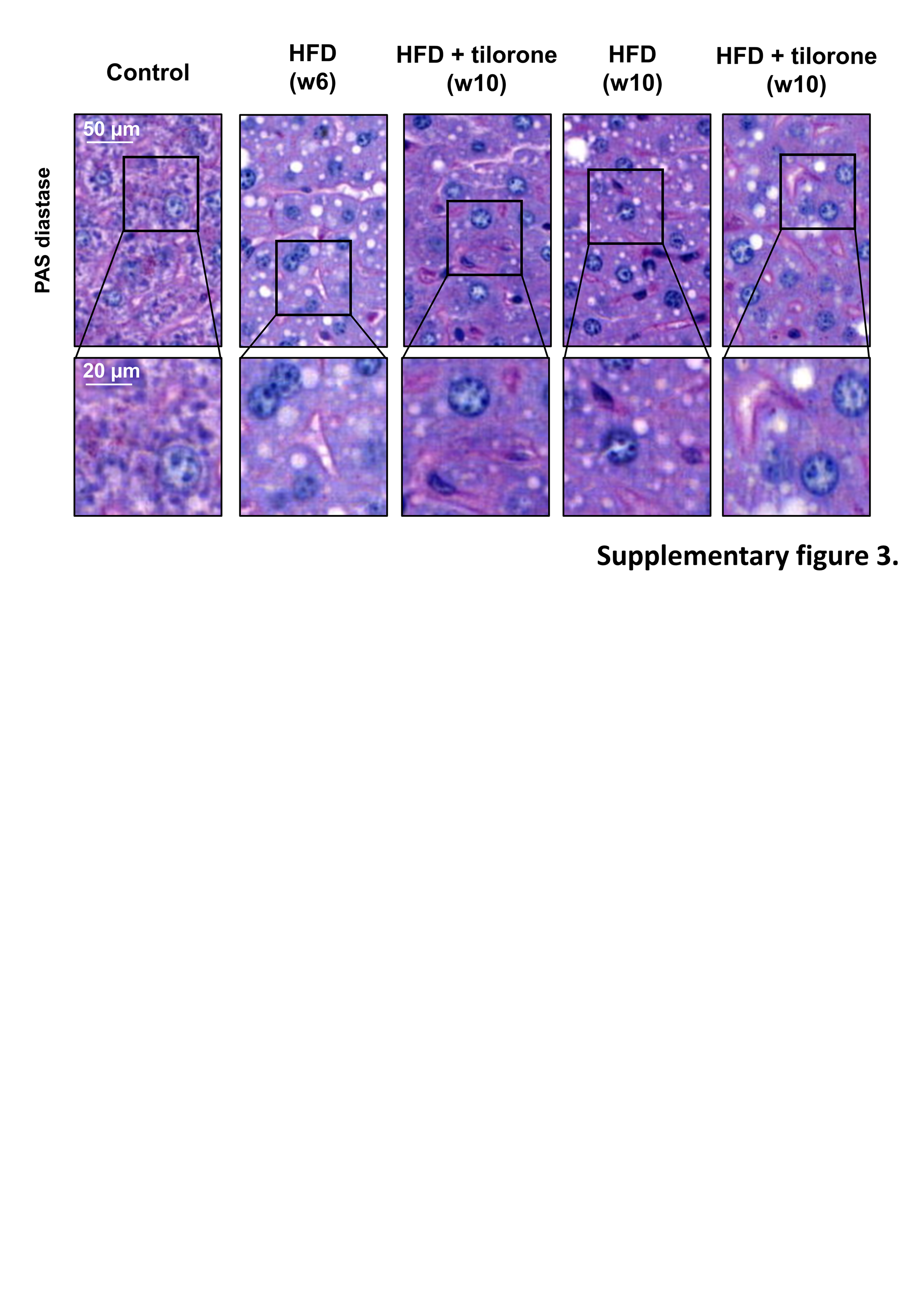

Supplement: Supplementary file 3 — Supplementary file3 (PNG 2009 KB) [file 11357_2025_1685_MOESM3_ESM.png]
